# Quality Changes in Live *Ruditapes philippinarum* During “Last Mile” Cold Chain Breakage: Effect of Packaging

**DOI:** 10.3390/foods14061011

**Published:** 2025-03-17

**Authors:** Yiming Huang, Xinrui Xie, Shoaib Younas, Caiyun Liu, Xin Wang

**Affiliations:** School of Health Science and Engineering, University of Shanghai for Science and Technology, Shanghai 200093, China; huangyiming19991114@126.com (Y.H.); xinrui1016@126.com (X.X.); shoaibfsn@yahoo.com (S.Y.); jnyzlcy@126.com (C.L.)

**Keywords:** *R. philippinarum*, cold chain breakage, last-mile delivery, packaging treatments, quality changes

## Abstract

The reliability of the “last mile” of cold-chain logistics is crucial for food safety. This study investigated the effect of different packaging treatments on the quality of anhydrously preserved live *Ruditapes philippinarum* (*R. philippinarum)* in “last mile” cold chain disruption. The temperature profiles of three packaging treatments at ambient temperature (25 °C) were monitored. Quality assessment was conducted based on sensory scoring, survival rate, total viable count (TVC), water-holding capacity (WHC), pH, total volatile basic nitrogen (TVB-N), thiobarbituric acid-reactive substances (TBA), color, and texture. Low-frequency nuclear magnetic resonance (LF-NMR) and magnetic resonance imaging (MRI) were utilized to characterize the water state profile. The findings demonstrated a progressive increase in internal package temperature throughout the “last mile”, with packages containing additional ice packs more effectively maintaining lower temperature and restricting the migration of “hot spots” towards the center. Specifically, the package with three ice packs maintained a markedly lower temperature, which effectively inhibited microbial activity, lipid oxidation, and the production of alkaline substances, resulting in higher survival rates, water-holding capacity, texture, sensory acceptability, and immobilized water fraction. Furthermore, LF-NMR relaxation parameters showed strong correlations with various physicochemical indices, suggesting a potential approach for real-time quality monitoring. This study provides insights for maintaining live *R. philippinarum* quality during the “last mile”.

## 1. Introduction

The consumption of shellfish has increased over time, accounting for 26% of aquatic animal food consumption in 2021 [1]. China boasts abundant live aquatic resources, yielding a total of 68.659 million tons in 2022, with live shellfish accounting for 23.86% of the total output [2]. Live shellfish have gained popularity due to their delicious taste [3] and rich nutritional value [4]. However, such products are highly perishable, geographically specific, and seasonal and have a short shelf life. The transportation of live shellfish products poses challenges in maintaining their survival rates and freshness. Furthermore, with the growing trend towards omni-channel retailing, e-commerce has become increasingly common for the sale of live aquatic products. This shift underscores the need for effective and scientifically grounded preservation and delivery methods for live shellfish.

Previous studies [5,6] have demonstrated that anhydrous preservation is a more cost-effective technique for live shellfish compared to water preservation, resulting in lower mortality rates and reduced nutrient loss [7]. It remains critical to maintain low temperatures during the delivery process, from producers to consumers, to preserve the freshness and quality of the shellfish [8]. For example, Bi et al. [9] found that anhydrous delivery at low temperature (4 °C) effectively maintains the high quality and physiological conditions of clams. Similarly, Jiang et al. [10] found that live *Patinopecten yessoensis* can be preserved for up to 5 days on ice. Thus, the cold chain logistics system plays a vital role in ensuring the quality and longevity of live shellfish products during distribution.

Currently, domestic delivery of live seafood during business-to-business (B2B) operations primarily relies on conventional refrigerated trucks [11]. This process has garnered significant attention, with numerous studies [12,13,14] focusing on improving refrigeration equipment and delivery technologies. However, in the business-to-consumer (B2C) process, frequent “cold chain breakage” occur due to the absence of professional transport equipment. Typically, only insulated containers and ice packs are used to maintain the cold chain. This challenge during the final stage of the delivery process, often referred to as the “last mile” phenomenon, is marked by temperature fluctuations caused by uncertainties in delivery time, delayed receipt, and a lack of cold storage facilities [15]. The distribution of temperature within the container is also influenced by the number and placement of ice packs. Such fluctuation is known to accelerate deterioration of live shellfish quality. For instance, Teng et al. [16] found that repeated freeze–thaw cycles significantly reduced the protein content, fat content, malondialdehyde (MDA) levels, sulfhydryl (-SH) levels, and Ca^2+^-ATPase activity in live pacific oysters. Therefore, maintaining a stable, low temperature field is vital for preserving the quality and freshness of the stored shellfish during “last mile” delivery [17,18]. In addition to traditional quality change investigation, analysis of temperature distribution is helpful for identifying the optimal placement and quantity of ice packs needed to maintain a stable, low temperature field. Although COMSOL Multiphysics^®^ has been successfully employed as the thermal modeling software package to study the “last mile” distribution method [19] based on temperature considerations, few studies have simulated the temperature field in the “last mile” for determining suitable packaging.

There has been no systematic study on the effects of temperature fluctuation during the “last mile” of the cold chain on live *R. philippinarum*. Therefore, this study aims to investigate quality changes in anhydrous-preserved live *R. philippinarum* during express delivery in the “last mile” of the cold chain. The live clams were preserved using different express packaging treatments. Firstly, COMSOL Multiphysics^®^ was used to simulate the temperature field in the box. Then, quality assessment was conducted based on sensory scoring, survival rate, TVC, and various physicochemical parameters, including WHC, pH, TVB-N, and TBA. Additionally, color analysis, texture profile, LF-NMR, and MRI were employed for further evaluation of quality alterations in live *R. philippinarum*. This study provides a reference for optimizing logistics distribution conditions and improving the quality of live *R. philippinarum*.

## 2. Materials and Methods

### 2.1. Materials and Reagents

Thiobarbituric acid, trichloroacetic acid, sodium chloride, boric acid, 1,1,2,3-tetraethoxypropane, and hydrochloric acid was purchased from Sinopharm Chemical Reagent Co., Ltd. (Shanghai, China). All materials were of at least analytical purity.

### 2.2. Sample Preparation

Live *Ruditapes philippinarum* (*R. philippinarum*) specimens (shell length: 40 mm, thickness: 5 mm) were purchased from the Oriental International Aquatic Center in Shanghai. After being placed in an ice container, they were transported to the laboratory within 1 h and stored at 4 °C. Live specimens with good activity, similar size, and other characteristics were selected from the same batch as the experimental samples. To simulate potential cold chain disruptions during business-to-consumer (B2C) transactions, four experimental groups were established. The samples were randomly assigned to four groups, each weighing 1000 g. Every 250 g portion was packaged in sterile polyethylene (PE) food-safe plastic bags.

One group was kept at 4 °C in a refrigerator as a control (CK), while the remaining three groups were each placed in an aluminum foil insulation bag with one, two, or three ice packs (dimensions: 150 × 105 × 2.5 mm). These insulated bags were then transferred to foam boxes (outer dimensions: 340 × 220 × 165 mm, internal dimensions: 300 × 180 × 145 mm) with foam lids (dimensions: 340 × 220 × 22 mm). The walls, lid, and base of the foam boxes all had the same thickness of 22 mm. The groups were labeled as R-1, R-2, and R-3 according to the number of ice packs used. The quality of the sample was assessed at four time points (0 h, 12 h, 24 h, and 48 h) at ambient temperature (25 °C).

### 2.3. Temperature Measurement

To monitor temperature changes during storage, a copper–constantan thermocouple probe (KEYSIGHT 34970, Keysight Technologies, Santa Rosa, CA, USA) was calibrated with an ice–water mixture prior to use. The probe was connected to a portable data logger and placed at the center of the clam shells. This method followed the protocol described by Manzocco et al. [20]. Monitoring continued until the samples were deemed inedible.

### 2.4. Modeling

#### 2.4.1. Physical Model

**Figure 1:**
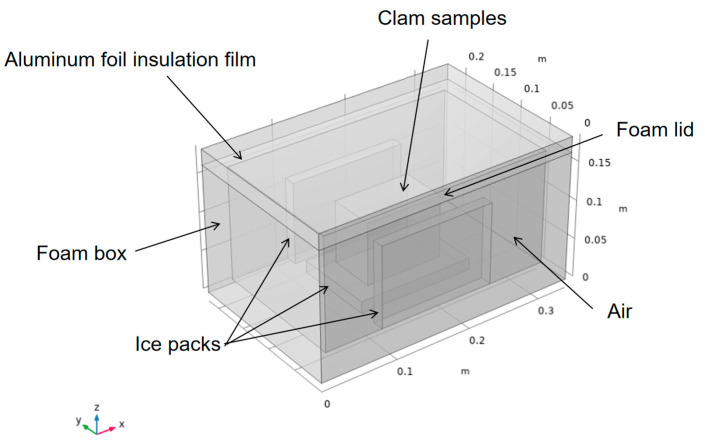
Physical geometry of the box.

Figure 1 presents the physical geometry of the foam box. The outer dimensions of the box were 340 mm (length) × 220 mm (width) × 165 mm (height), while the internal dimensions were 300 mm (L) × 180 mm (W) × 145 mm (H). The thermostat requires insulation, such as foam, to separate the contents from the outside environment. To reduce heat transfer, aluminum foil insulation was then added to form a composite insulation layer with foam. This was followed by the addition of a source of cold, such as ice packs at −5 °C, which were placed vertically around the contents (clams). The remaining space in the interior was filled with air. The initial temperature of the contents was 5 °C.

#### 2.4.2. Assumptions and Governing Equation

To simplify the modelling process, the following assumptions were made:

(1) All materials are considered isotropic.
(2) The air inside the foam box is regarded as an incompressible fluid.
(3) The initial temperature of the ice packs and the box interior is assumed to be uniform.
(4) Changes in the density of the ice packs are neglected, thus assuming a constant volume for the ice packs [21].

The primary modes of heat transfer in the foam box include natural convection of the air inside the box and heat conduction between the internal components. In the context of cold food storage, the latent heat of phase change in the ice packs should also be considered.

The heat transfer within the solid and liquid phases follows the energy balance Equations (1) and (2):

(1) $$\rho C_{P}\frac{\partial T}{\partial t}+\rho C_{P}u\cdot \nabla T+\nabla \cdot q=Q$$

(2) q=−k∇T

The model fluid heat transfer Equations (3) and (4) are as follows:

(3) $$\rho C_{P}\frac{\partial T}{\partial t}+\rho C_{P}u\cdot \nabla T+\nabla \cdot q=Q+Q_{P}+Q_{vd}$$

(4) q=−k∇T

where ρ, $C_{P}$, *T*, *u*, *q*, *Q*, $Q_{P}$, and $Q_{vd}$ are the density, specific heat capacity, temperature, external field dependent variable, heat flux, heat flow generated internally, internal energy, and kinetic energy, respectively. Because of the small effect of natural convection and the incompressible nature of air, $Q_{P}$ and $Q_{vd}$ are negligible.

In addition, phase transition heat transfer is also involved. The phase change heat transfer Equations (5)–(9) are as follows:

(5) $$\rho =\theta_{phase1}\rho_{phase1}+{\theta_{phase2}\rho }_{phase2}$$

(6) $$C_{p}=\frac{1}{\rho }(\theta_{phase1}\rho_{phase1}C_{p,phase1}+\theta_{phase2}\rho_{phase2}C_{p,phase2})+L_{phase1\to phase2}\frac{\partial \alpha_{m}}{\partial T}$$

(7) $$\alpha_{m}=\frac{1}{2}\frac{\theta_{phase2}\rho_{phase2}-\theta_{phase1}\rho_{phase1}}{\theta_{phase1}\rho_{phase1}+\theta_{phase2}\rho_{phase2}}$$

(8) $$k=\theta_{phase1}k_{phase1}+\theta_{phase2}k_{phase2}$$

(9) $$\theta_{phase1}+\theta_{phase2}=1$$

where the subscript symbols *phase1* and *phase2* are liquid water and solid water (ice), respectively. θ, ρ, $C_{p}$, and k are a percentage, the density, the specific heat capacity at constant pressure, and the thermal conductivity, respectively. The parameters for the phase change material (ice packs) used in the model, as provided by Perrot [22], are given in Table 1. The samples placed in the box are clams. Table 2 presents the characteristics of the clams.

#### 2.4.3. Initial Conditions and Boundary Conditions

The box walls consisted of insulation materials, and the ice packs were in direct contact with the internal surfaces of the box. The air temperature outside the box was constant, at 25 °C. The heat transfer boundary condition between the box surface and the air was set as natural convection with a convection heat transfer coefficient of 5 W·m^−2^·K^−1^, which was calculated using the following Equation (10):

(10) $$Q=hA(T\;-\;T_{0})$$

where *Q* is the amount of heat exchanged between the clams and the atmosphere per unit time (W); *h* is the convective heat transfer coefficient (W·m^−2^·K^−1^); *A* is the surface area of the boundary between the food and the atmosphere (m^2^); *T* is the temperature of the food surface (°C), and *T_0_* is the ambient temperature (°C). The box enclosure was set as thermal insulation.

The heat is transferred through the wall to the product at the bottom and then through the height of the product to the ice, causing it to melt and thereby inducing a phase change [24]. Under the relevant assumptions, the phase change heat transfer problem of water melting is simplified to a transient heat transfer problem, which only involves the first kind and third kind of boundary conditions.

#### 2.4.4. Geometric Model Meshing and Numerical Solution

The box was nearly cuboid; thus, this shape was considered to simulate the geometric model of the box. A finite element method was employed to solve the transient heat transfer problem, enabling the calculation of temperature–time histories within the three-dimensional box model. The numerical solution was obtained using COMSOL Multiphysics Software 6.2. The model mesh was divided before model computing. A physics-controlled mesh consisting of tetrahedral area elements was generated for the simulations. For phase change materials, “finer” meshes must be used to accurately simulate the melting front. To reduce the amount of computer calculation and shorten the calculation time, other domains were set as “fine” tetrahedral meshes. The final grid system consisted of 57,481 domain elements (tetrahedral), 10,390 boundary elements (triangular), 916 edge elements (linear), and 60 vertex elements. The heat transfer inside the box varies with time, which requires instantaneous solving. In addition, the total time of the solution, initial time step, and relative tolerance were 48 h, 1 h, and 0.001, respectively. The server configuration for computing was a Lenovo processor, 13th Gen Intel (R) Core (TM) i9-13900HX, 2200 Mhz, 32 GB RAM, and a Win11 Server Standard 64-bit operating system.

#### 2.4.5. Verification of Heat Transfer Model

Numerical and experimental results were compared to evaluate the accuracy of the model. One measure of forecast consistency, i.e., the root mean squared error (RMSE), was used to assess the reliability and accuracy of the heat transfer model [25], which can be calculated using the following Equation (11):

(11) $$RMSE=\sqrt{\frac{1}{n}\sum_{i=1}^{n}{\left(Q_{i}-P_{i}\right)}^{2}}$$

where *Q_i_* and *P_i_* are the values of observed and predicted temperatures (°C); n is the number of observations; and *Q_i_* is the average of observed values. Values of RMSE close to 0 are considered to indicate better model prediction.

### 2.5. Sensory Evaluation

Sensory evaluations were conducted according to the method of Manousaridis et al. [26], with slight modifications. The sensory panel consisted of eight trained members. The results were recorded using a 10-point scale across four categories: color (0: dull, 10: milky white), smell (0: spoilage odor, 10: no off-odor), appearance (0: flesh detachment, 10: intact), and texture (0: high viscosity, 10: firm). The scores from these categories were averaged to calculate the overall “sensory score” (ranging from 0 to 10), and the acceptability was determined as having a score of over 6. Data were compiled from the independent evaluations of the eight panel members and presented as the mean score.

### 2.6. Survival Rate Assessment

Survival rate was measured according to the method of Gonçalves et al. [27], where the survival of clams was determined by observing their valve responses. Clams with open shells were gently tapped with a glass rod to monitor their reactions. The clam was considered dead if its shell remained open for an extended period. The survival rate was calculated using Equation (12):

*Survival rate (%) = (live animals/total animals)* × *100* (12)

### 2.7. TVC Measurements

The total viable count was determined using the method described by Alcicek [28]. Live clams were initially selected, and a 10 g sample of clam flesh was homogenized with 90 mL of sterile saline. After homogenization, the mixture underwent 10-fold serial dilutions, with each dilution prepared in triplicate. From each dilution, 100 μL was plated onto the agar plates (PCA) and incubated aerobically at 30 ± 1 °C for 72 ± 3 h. The bacterial population on each plate was counted and expressed as logarithmic colony-forming units per gram (log CFU/g).

### 2.8. Determination of Physicochemical Properties

#### 2.8.1. WHC Measurements

WHC was assessed by measuring cooking loss and drip loss. Cooking loss was determined based on the method of Wang et al. [29], with slight modifications. A 5 ± 0.1 g sample of clam flesh was heated at 80 °C for 5 min, then cooled and weighed. Each sample group was in triplicate, and cooking loss was calculated using Equation (13):

(13) $$Cooking\;loss\;(\%)=\frac{m_{0}\;-m_{1}}{m_{0}}\times 100$$

where $m_{0}$ and $m_{1}$ represent the weight of the sample before and after cooking, respectively.

Drip loss was determined according to the modified method of Gudjónsdóttir et al. [30]. A 2 ± 0.1 g sample of clam flesh was placed in a 15 mL centrifuge tube with two layers of filter paper at the bottom. The samples were centrifuged at 1500 rpm for 10 min using a KA-1000 centrifuge (Shanghai Anting Scientific Instrument Factory, Shanghai, China), and the weight was recorded. Drip loss rate was calculated according to Equation (14):

(14) $$\mathrm{Drip}\;\mathrm{loss}\;(\%)=\frac{m_{2}-m_{3}}{m_{2}}\;\times 100$$

where $m_{2}$ and $m_{3}$ represent the weight of the sample before and after centrifugation, respectively.

#### 2.8.2. pH Measurements

The pH was measured according to the method of Liu et al. [31], with slight modifications. A 10 ± 0.1 g sample of clam flesh was mixed with 100 mL of distilled water and allowed to stand for 30 min. The pH of the resulting filtrate was then determined using a pH meter (S210K, Mettler Toledo International Ltd., Zurich, Switzerland).

#### 2.8.3. TVB-N Measurements

TVB-N was measured according to the method of Sun et al. [32], with slight modifications. A 10 g sample of clam flesh was homogenized with 75 mL of distilled water, followed by the addition of 1.0 g of MgO. The mixture was processed in an automatic Kjeldahl apparatus (K1100, Hanon Advanced Technology Group Co., Ltd., Jinan, China) for nitrogen determination. The liquid was collected in a bottle containing 25 mL of a 2% (*m*/*v*) boric acid solution, which was subsequently titrated with 0.0101 mol/L hydrochloric acid (HCl) until a pink endpoint was achieved. The volume of HCl used was recorded and the TVB-N content was calculated using Equation (15):

(15) $$\mathrm{TVB}-N/(\mathrm{mg}/100\;\mathrm{mL})=\frac{{(V}_{1}-V_{2})\times c\times 14}{m}\times 100$$

where $V_{1}$ was the volume of HCl consumed by the samples, $V_{2}$ was the volume of HCl consumed by the blank, *c* was the concentration of HCl, and m was the mass of the samples.

#### 2.8.4. TBA Measurements

TBA was measured following the method of Liu et al. [33]. A 5 g sample of ground clam flesh was mixed with 50 mL trichloroacetic acid and shaken for 30 min at 0 °C, then filtered. Subsequently, 5 mL of the filtrate was mixed with 5 mL of thiobarbituric acid (TBA) solution and heated at 90 °C for 30 min. After cooling, the absorbance of the supernatant was measured at 532 nm using a UV 9100D spectrophotometer (LabTech, Inc., Hopkinton, MA, USA). A standard curve was prepared using 1,1,2,3-tetraethoxypropane, and the absorbance values were used to calculate the TBA content.

### 2.9. Color Analysis

Referring to the method of Chen et al. [34], color measurements, including lightness (L*), redness (a*), and yellowness (b*), were determined using a chromameter (CR-400, Chroma Japan Corp., Yokohama, Japan). The instrument was calibrated with a white ceramic plate prior to measurement.

### 2.10. Texture Measurements

After removing the shells and blotting excess moisture from the surface of the clam flesh, the texture profile of the samples was analyzed according to the method described by Yang at al. [35], with slight modifications. The texture characteristic parameters (hardness, viscosity, elasticity, cohesiveness, chewiness, and resilience) were determined using a TA-XTplus texture analyzer (Stable Micro Systems Ltd., Godalming, UK) equipped with a P/50 flat-bottomed cylindrical probe in TPA mode. The measurement settings were as follows: test speed of 1 mm/s, pre-test and post-test speeds of 2 mm/s, and a deformation rate of 60%. Each sample was tested at least eight times, and the average value was calculated.

### 2.11. LF-NMR and MRI Measurements

#### 2.11.1. LF-NMR Measurements

LF-NMR was performed according to the method of Wang et al. [36], with minor modifications. A PQ-001 NMR analyzer (Shanghai Niumag Analytical Instrument Co., Shanghai, China) with a magnetic field strength of 0.5 T and a proton resonance frequency of 21.3 MHz was employed. A 5.0 ± 0.01 g sample of minced clam flesh was transferred to a 15 mm NMR tube and equilibrated to 35 °C before measurement. Transverse relaxation (T_2_) was determined using the Carr–Purcell–Meiboom–Gill pulse sequence (CPMG). Data were collected from 2000 echoes across eight scans, with a repetition time of 4500 ms between scans and an interval of 300 μs between the 90° and 180° pulses.

T-invfit software V2.1, based on Simultaneous Iterative Reconstruction technique (SIRT), was used to obtain the single component relaxation time (T_2W_) and the transverse relaxation time (T_2_) distribution through mono-exponential or multi-exponential fitting. The peak area (A_2i_), normalized peak proportion (S_2i_), and transverse relaxation time (T_2i_) were obtained from the transverse relaxation time (T_2_) distribution.

#### 2.11.2. MRI Measurements

Proton density imaging of the samples was conducted using MRI, following the parameters outlined by Cheng et al. [37]. T_2_ weighted images were obtained using a spin-echo (SE) imaging sequence with the following settings: field of view (FOV) = 80 mm × 80 mm, three slices with a slice thickness of 1.5 mm, repetition time (TR) = 500 ms, and echo time (TE) = 20 ms. Shelled samples were placed in a nuclear magnetic tube, and adjustments were made to the signal-to-noise ratio and image clarity to obtain optimal imaging. The resulting grayscale images were then subjected to pseudo-color processing to enhance visual interpretation.

### 2.12. Statistical Analysis

Each experiment was conducted in triplicate, with results expressed as the mean ± standard deviation. For statistical analysis, one-way analysis of variance (ANOVA) was performed using SPSS 18.0 (SPSS Inc., Chicago, IL, USA). Duncan’s test was applied at a 95% confidence level (*p* < 0.05), and Pearson correlation analysis was carried out to examine relationships between variables. Data fitting and plotting were performed using Origin 2023 software.

Pearson correlation analysis was used to explore the relationship between LF-NMR results and physicochemical parameters. In this analysis, the effects of the treatments were not considered, and the mean values across all treatments were used.

## 3. Results and Discussion

### 3.1. Temperature Distribution and Validation of the Model

**Figure 2:**
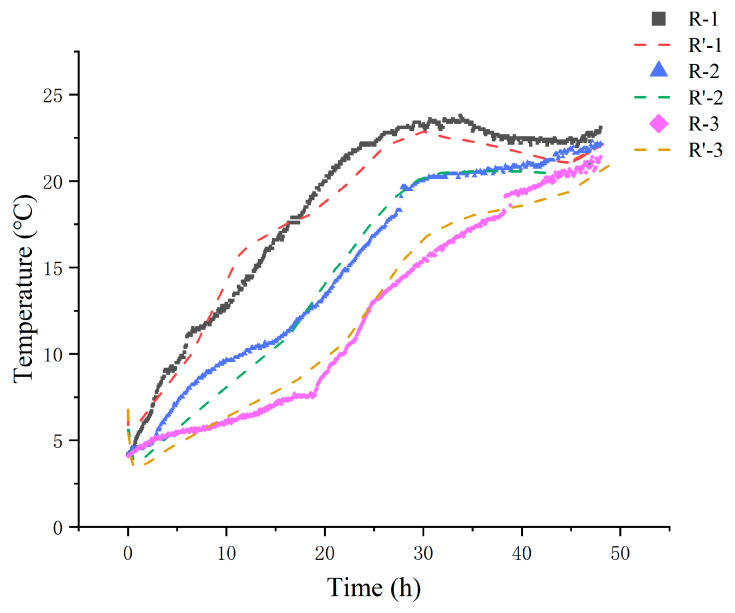
Comparison of experimental (R-) and simulated (R’-) internal temperatures of different treatments. R-1 is the group with one ice pack, R-2 is the group with two ice packs, and R-3 is the group with three ice packs.

The National Shellfish Sanitation Program (NSSP) recommends that live shellfish should be stored in an environment kept at or below 7.2 °C using ice or mechanical refrigeration [38]. Monitoring temperature fluctuations during delivery is, therefore, crucial. The temperatures of live clams in the R-1, R-2, and R-3 groups were recorded throughout the delivery process and compared to the simulated values (Figure 2). The internal temperature, initially at 5 °C, increased progressively over time. In the R-1 and R-2 groups, which were supplied with fewer ice packs, the temperature rose rapidly, surpassing the recommended threshold by reaching 7.4 °C within 3 h and 7.2 °C within 5 h. Conversely, the R-3 group, with more ice packs, only reached 7.3 °C after 15 h. As the process continued up to 48 h, temperature stabilization occurred, which led to a reduced temperature differential. By the end of the 48 h period, the measured temperatures of the R-1, R-2, and R-3 groups were 23.1 °C, 22.2 °C, and 21.4 °C, respectively.

A comparison between the simulated and experimental values (Figure 2) shows strong alignment. Across all groups, the temperature difference between the experimental and simulated results was generally less than 1.5 °C. This agreement was further quantified by the RMSE coefficient at different times (Table 3), which was within the range of 0.10–0.97 °C, with a gradual decrease observed over time. Slight deviations are observed in the temperature curves during the phase transition period of the ice packs, which is consistent with the research by Pattanaik and Jenamani [39]. Analysis of unstable natural convection is subject to disturbances and boundary condition settings. Factors such as airflow and heat conduction cause non-uniform heat transfer, condensation, deformation, and changes in ice pack contact conditions with the insulation material. Overall, the results indicate that the heat transfer model well represented the temperature distribution in the box with satisfactory accuracy.

**Figure 3:**
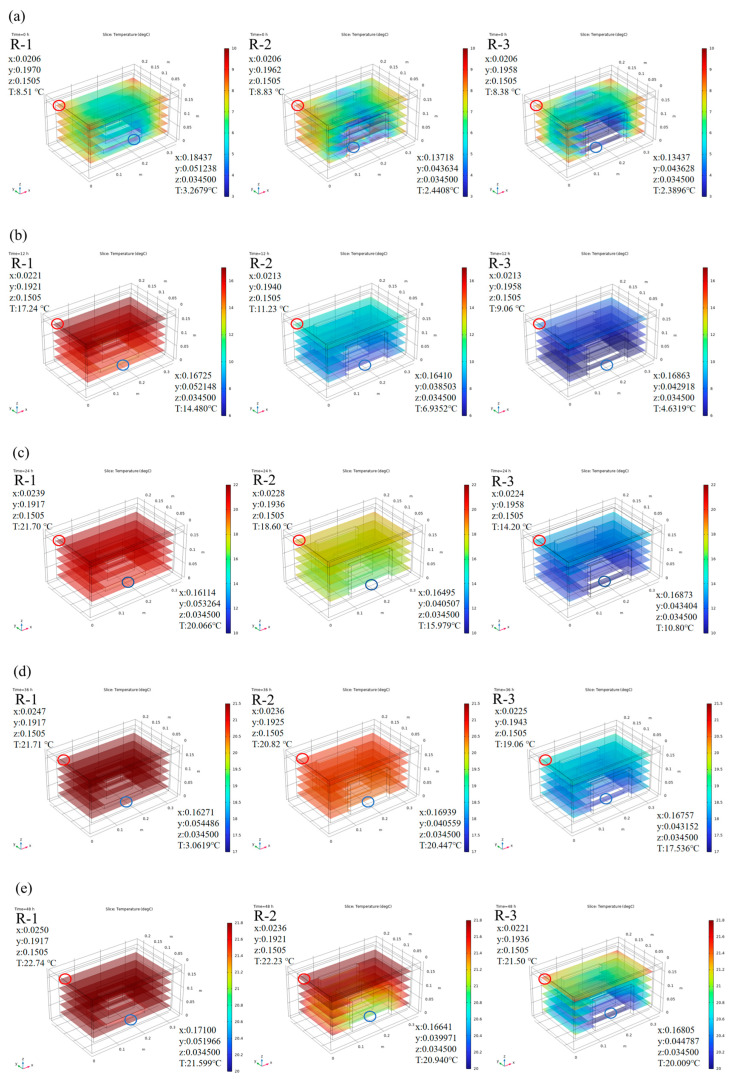
Predicted internal temperature distribution of different groups at 0 h (**a**), 12 h (**b**), 24 h (**c**), 36 h (**d**), and 48 h (**e**). R-1 is the group with one ice pack, R-2 is the group with two ice packs, and R-3 is the group with three ice packs. The red circle indicates the location of hot spots, and the blue circle indicates the location of cold spots.

Based on the model calculations, we analyzed the effect of different packing schemes. Figure 3 shows the predicted temperature distribution in the R-1, R-2, and R-3 groups during cold chain breakage, where red presents higher temperatures and blue indicates lower temperatures. Considering that all ice packs were positioned near the bottom of the box, slightly lower temperatures at the bottom were observed due to direct contact between the samples and the ice packs. As expected, the addition of more ice packs resulted in greater cold air release, leading to lower temperatures at any given time point. The R-1 group, with only one ice pack positioned at the bottom, exhibited the highest temperatures among all groups. The temperature difference between the groups was most pronounced within the first 24 h. The highest temperature area was located at the top interior of the container in the R-1 group, and the lowest temperature area was located at the bottom in the R-3 group. A maximum difference of 10 °C was observed between the R-1 (21.7 °C) and R-3 (10.8 °C) groups at the 24 h mark. As the temperature changes in each group began to stabilize, the temperature gap narrowed; this trend was consistent with the earlier temperature curves.

Meanwhile, the temperature distribution within the container was not uniform, and “hot spots” were noticed, which were further investigated to understand their behavior. Due to the regular symmetry of the model, hot spots were evenly distributed in the four corners of the top interior of the container. For detailed analysis, one corner was taken as a representative example. In the R-1 group, the temperature in the hot spot increased to 21.70 °C at 24 h, while that for the R-3 group was only 14.20 °C. Movement of hot spots over time was also noted. For instance, in the R-1 group, the hot spot initially started in the corner with coordinates 0.0206, 0.1970, 0.1505 and a temperature of 8.51 °C. It gradually shifted toward the center and eventually reached 22.74 °C at coordinates 0.0250, 0.1917, 0.1505 by 48 h. In comparison, the hot spots of the groups with fewer ice packs were positioned closer to the center, while in groups with more ice packs, the hot spots remained near the corners. For example, the hot spots in the R-1, R-2, and R-3 groups were located at 0.0247, 0.1917, 0.1505; 0.0236, 0.1925, 0.1505; and 0.0225, 0.1943, 0.1505, with corresponding temperatures of 21.71 °C, 20.82 °C, and 19.06 °C at 36 h, respectively.

The simulation results provide valuable insights into the temperature distribution of the box, which is critical for optimizing the transportation conditions of live *R. philippinarum*. By analyzing the temperature field of the box, it is evident that the addition of more ice packs can effectively maintain lower temperatures and restrict the movement of hot spots toward the center, therefore improving uniformity of temperature distribution. This is essential for maintaining the viability and quality of the fresh product.

### 3.2. Sensory Analysis

**Figure 4:**
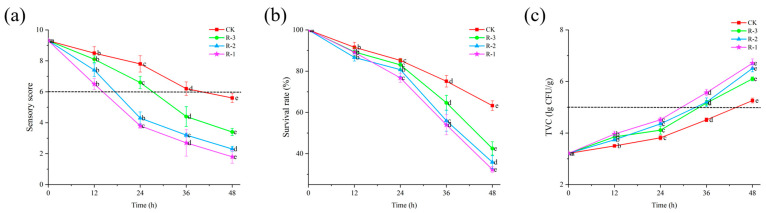
Effects of different packaging treatments on the sensory score (**a**), survival rate (**b**), and TVC (**c**) of live clams during delivery. Different letters indicate significant differences between different times for the same treatment (*p* < 0.05). CK is the control group, which was stored at 4 °C. R-1 is the group with one ice pack, R-2 is the group with two ice packs, and R-3 is the group with three ice packs, all of which were stored at 25 °C.

Sensory evaluation serves as the most intuitive indicator of food freshness [40]. As shown in Figure 4a, the sensory scores of live *R. philippinarum* in each group decreased significantly with the extension of cold chain breakage (*p* < 0.05). The R-1 group exhibited the most rapid decline in sensory scores, while the R-3 group showed a slower decline. Compared to the CK and R-3 groups, both of which maintained sensory scores above 8, the sensory scores in the R-1 and R-2 groups dropped to 7.4 and 6.5, respectively, after 12 h. At 24 h, the sensory scores in the R-1 and R-2 groups dropped below the rejection limit, reaching 3.8 and 4.3, respectively. As delivery extended, even the CK group, which was stored at 4 °C, experienced a decline in sensory scores below the sensory rejection limit, reaching 5.6 at 48 h. At that time, the R-3 group still exhibited a sensory score of 3.4, while rapid deterioration was observed in the R-1 and R-2 groups, with significantly low scores of 1.8 and 2.3, respectively (*p* < 0.05). The sensory quality of live *R. philippinarum* deteriorated to varying degrees due to the action of enzymes and microorganisms [41], and temperature fluctuations can further accelerate deterioration of the sensory quality of clams. These results align with findings by Li et al. [42], who reported that low temperatures can better maintain the overall sensory freshness of razor clams.

### 3.3. Survival Rate

The survival rate can directly reflect the overall freshness of *R. philippinarum*. As shown in Figure 4b, the initial survival rate of *R. philippinarum* in all groups was 100%. With delivery time extension, the survival rate exhibited a gradual decline initially, followed by a more rapid decrease in the later stages (*p* < 0.05), with higher mortality observed in groups with fewer ice packs. This indicates that a slow change of temperature is beneficial for improving the survival rate of aquaculture products [43]. During the first 12 h, there was no significant difference in survival rates among the groups. By the 24 h mark, the survival rates of the CK and R-3 groups remained above 83%, whereas those of the R-1 and R-2 groups dropped to 76.72% and 80.72%, respectively. As time progressed, increasing temperatures stimulated the activity of spoilage bacteria and enzymes, accelerating mortality of the shellfish [44]. After 48 h, the survival rates of the R-1 and R-2 groups, which had fewer ice packs and were exposed to higher temperatures, dropped significantly, to 32.33% and 35.84%, respectively.

### 3.4. Microbiological Analysis

Microbial proliferation is a major factor contributing to the spoilage of aquatic products. According to the U.S. Food and Drug Administration (FDA) microbial standard [45], the threshold for Total Viable Count (TVC) in shellfish is set at 5 × 10^5^ CFU/g. As illustrated in Figure 4c, the TVC in all four groups of *R. philippinarum* exhibited a continuous increase throughout the delivery period. Compared to the initial TVC for the sample, that is 3.21 lg CFU/g, the TVC of the CK reached 3.82 log CFU/g after 24 h, while the R-1, R-2, and R-3 groups showed slightly higher values of 4.51 log CFU/g, 4.35 log CFU/g, and 4.11 log CFU/g, respectively. As the delivery time progressed, internal temperatures within the packaging gradually increased, which created favorable conditions for bacterial growth. This result is consistent with the findings of Fernandez-piquer et al. [46], who reported that high temperatures during live oyster storage can lead to an increase in TVC, decreasing product shelf life. By the 48 h mark, the TVC levels had increased significantly in all groups, reaching 5.26, 6.71, 6.51, and 6.10 log CFU/g for the CK, R-1, R-2, and R-3 groups, respectively. All values exceeded the FDA limit of 5 × 10⁵ CFU/g. Notably, the R-1 group, which contained fewer ice packs, recorded the highest TVC at 6.71 log CFU/g, surpassing the other groups. Bernardez and Pastoriza [47] reported that elevated temperatures, along with the accumulation of waste or decomposition products from dead mussels within containers, promote microbial growth. This finding aligns with our survival rate results. Furthermore, these outcomes are consistent with the study by Love et al. [48], which demonstrated that cold chain management can effectively inhibit the growth of *Vibrio parahaemolyticus* in live oysters.

### 3.5. Physical and Chemical Analysis

#### 3.5.1. WHC

**Figure 5:**
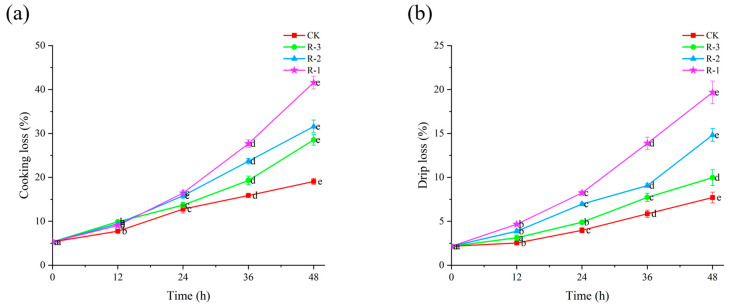
Effects of different packaging treatments on cooking loss (**a**) and drip loss (**b**) of live clams during delivery. Different letters indicate significant differences between different times for the same treatment (*p* < 0.05). CK is the control group, which was stored at 4 °C. R-1 is the group with one ice pack, R-2 is the group with two ice packs, and R-3 is the group with three ice packs; all were stored at 25 °C.

Cooking loss and drip loss can reflect the ability of shellfish to retain their intrinsic water content during delivery [49]. Figure 5 shows the cooking and drip loss of the four groups. Initially, the cooking loss and drip loss for live samples were measured at 5.32% and 2.16%, respectively. With the extension of delivery time, both cooking loss and drip loss increased significantly (*p* < 0.05). As seen in Figure 5a, within the first 24 h, cooking loss in the CK (3.97%) and R-3 (4.89%) groups remained below 5%, while the R-1 and R-2 groups recorded higher values of 8.23% and 6.95%, respectively. However, cooking loss rose sharply to 19.07%, 41.63%, 31.58%, and 23.53% for the CK, R-1, R-2, and R-3 groups at 48 h, respectively (*p* < 0.05). Among the groups, R-1, which contained fewer ice packs, experienced the most rapid temperature increase, resulting in the highest cooking loss. The rise in temperature during delivery contributed to structural degradation of muscle cells, leading to increased water loss and a consequent decline in overall quality [50].

As shown in Figure 5b, the trends for drip loss followed a pattern similar to that observed for cooking loss. The CK group exhibited relatively lower drip loss, while the R-1 group showed the highest and most rapid increase. In the first 12 h, the drip loss in all groups remained below 5%. However, the drip loss in the R-1 and R-2 groups increased significantly to 19.67% and 14.83% after 48 h delivery, respectively, while that for the CK and R-3 groups remained under 10%. This sharp increase in drip loss is likely due to the reduction in ice packs leading to a rise in temperature, which in turn significantly accelerated proteolysis and degradation of the muscle structure, leading to tissue collapse and a subsequent decline in WHC [51,52].

#### 3.5.2. pH

**Figure 6:**
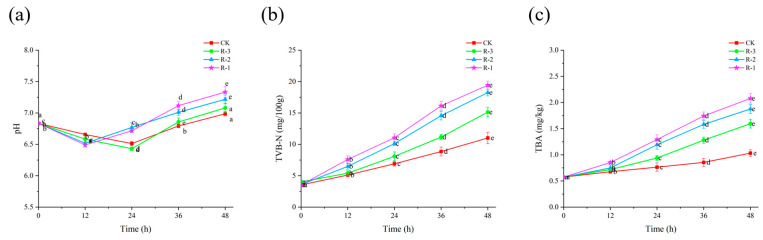
Effects of different packaging treatments on the pH (**a**), TVB-N (**b**), and TBA (**c**) of live clams during delivery. Different letters indicate significant differences between different times for the same treatment (*p* < 0.05). CK is the control group, which was stored at 4 °C. R-1 is the group with one ice pack, R-2 is the group with two ice packs, and R-3 is the group with three ice packs; all were stored at 25 °C.

Microbial growth and subsequent protein degradation lead to an increase in pH, making it a valuable indicator of the freshness of *R. philippinarum* [53]. As shown in Figure 6a, the initial pH of live *R. philippinarum* was 6.83, followed by a slight decline before rising again as delivery time extended. The inflection points of pH changes varied between groups. The pH values of the R-1 and R-2 groups reached their lowest values (6.49 and 6.52, respectively) at 12 h, but then rapidly increased to 7.33 and 7.22 by 48 h. In contrast, the lowest pH values for the CK and R-3 groups appeared at 24 h (6.51 and 6.43), and their pH values increased more slowly, reaching 6.99 and 7.08 at 48 h, respectively. This can be explained by the use of more ice packs ensuring lower temperatures throughout cold chain logistics, therefore suppressing the production of alkaline substances from microbial activity [54].

#### 3.5.3. TVB-N

Protein degradation leads to the accumulation of volatile basic nitrogenous compounds, and TVB-N is a reliable indicator of freshness in aquatic products [55]. As shown in Figure 6b, the initial TVB-N value of the live *R. philippinarum* samples was only 3.54 mg/100 g. With the extension of delivery time, the TVB-N levels increased significantly (*p* < 0.05), showing a slow rise initially followed by a rapid increase during the later stages of delivery. In the first 12 h, no significant differences were observed among the groups, with all TVB-N values remaining under 8 mg/100 g. This observation aligns with findings from [56], which reported that low temperatures limit the accumulation of TVB-N by inhibiting microbial activity and reducing enzyme activity. Consequently, only small amounts of amines were produced during this period. However, after 36 h, the R-1 group exhibited the highest TVB-N levels, reaching 16.14 mg/100 g. By 48 h, the TVB-N values for the CK, R-1, R-2, and R-3 groups were 11.02, 19.42, 18.25, and 15.13 mg/100 g, respectively. Notably, all groups except CK exceeded the national standard for marine shellfish (TVB-N ≤ 15 mg/100 g) [57]. This rise in TVB-N is largely attributable to metabolites produced by spoilage bacteria that induce protein degradation. Although low temperatures may not completely prevent the increase in TVB-N, they can significantly slow bacterial proliferation and delay protein degradation, as observed in [58].

#### 3.5.4. TBA

TBA is a key indicator of lipid oxidation [59]. Compared to the initial TBA value of live *R. philippinarum* (0.57 mg/kg), shown in Figure 6c, the TBA values of all the groups all increased along with extension of the delivery period (*p* < 0.05). Within the first 24 h, the TBA values in the R-1 and R-2 groups had already surpassed 1 mg/kg, reaching 1.29 mg/kg and 1.19 mg/kg, respectively. In contrast, the CK and R-3 groups exhibited lower TBA values of 0.76 mg/kg and 0.94 mg/kg. At 48 h, the TBA values of the CK, R-1, R-2, and R-3 groups reached 1.31, 2.08, 1.88, and 1.59 mg/kg, respectively, all of which exceeded the critical threshold for spoilage in aquatic products (1.0 mg/kg) [60]. Studies on mussels (*Perna canaliculus*) [61] and Pacific oysters (*Crassostrea gigas*) [62] have shown that elevated temperatures exacerbate unsaturated fatty acid metabolism, leading to accelerated lipid oxidation and the development of off-odors.

### 3.6. Color Measurements

**Figure 7:**
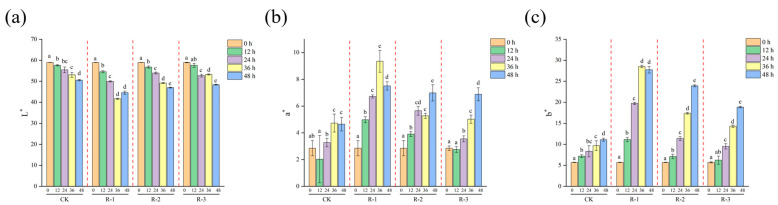
Effects of different packaging treatments on the color parameters L* (**a**), a* (**b**), and b* (**c**) during delivery. Different letters indicate significant differences between different times for the same treatment (*p* < 0.05). CK is the control group, which was stored at 4 °C. R-1 is the group with one ice pack, R-2 is the group with two ice packs, and R-3 is the group with three ice packs; all were stored at 25 °C.

Color is a key quality attribute that consumers often associate with taste and overall product quality [63]. As presented in Figure 7, a significant decline in L* values (*p* < 0.05) was observed with prolonged delivery time, alongside a significant increase in a* and b* values (*p* < 0.05). Overall, the color differences in the R-1 and R-2 groups were more pronounced during the delivery period, whereas the CK and R-3 groups showed minimal color variation, indicating that having fewer ice packs exacerbated the color changes. Specifically, the L* value in the R-1 group significantly decreased from 58.91 to 41.65, while the a* value increased from 2.85 to 9.35 and the b^*^ value increased sharply from 5.69 to 28.51 after 36 h. In contrast, the R-3 group exhibited more stable color changes, with L* decreasing to 48.37, and the a* and b* values increasing to 6.88 and 18.85 at 48 h, respectively. Color changes are influenced by pigment activity, protein denaturation, the presence of bioactive compounds [64], and hormonal regulation related to environmental stress [65], which can be accelerated by elevated temperatures; therefore, maintaining a low-temperature environment effectively inhibited color changes in live *R. philippinarum*.

### 3.7. Texture Properties

**Figure 8:**
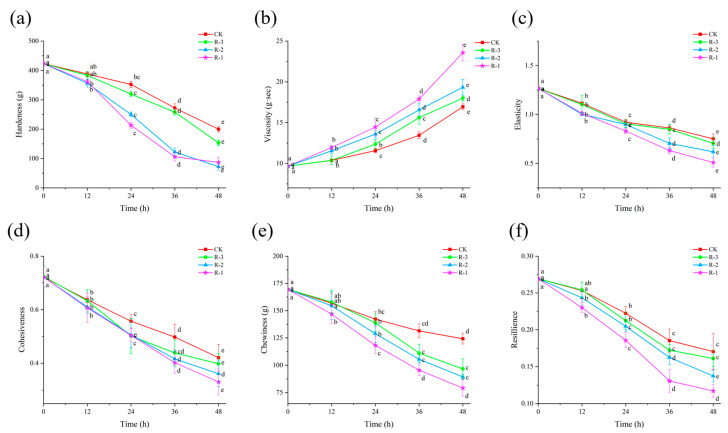
Effects of different packaging treatments on the texture properties of hardness (**a**), viscosity (**b**), elasticity (**c**), cohesiveness (**d**), chewiness (**e**), and resilience (**f**) during delivery. Different letters indicate significant differences between different times for the same treatment (*p* < 0.05). CK is the control group, which was stored at 4 °C. R-1 is the group with one ice pack, R-2 is the group with two ice packs, and R-3 is the group with three ice packs; all were stored at 25 °C.

As shown in Figure 8, the hardness, elasticity, cohesiveness, chewiness, and resilience of the samples significantly decreased (*p* < 0.05), while viscosity significantly increased (*p* < 0.05), as delivery progressed. The higher the sample temperature, the more change occurred in texture properties. Groups with higher internal temperatures exhibited more pronounced changes in these textural parameters.

Hardness, in particular, is a key index reflecting the internal binding force that allows food to maintain its structure [66]. As shown in Figure 8a, compared with the initial hardness value of the sample (423.29 g), the decrease in hardness was minor during the first 12 h, with all groups maintaining values above 350 g. However, a rapid decline occurred thereafter, especially the hardness in the R-1 and R-2 groups, which significantly fell below 250 g at 24 h, with R-3 maintaining a slightly higher value of 319.41 g. After 48 h, the hardness values in the CK, R-1, R-2, and R-3 groups dropped significantly to 199.73, 76.84, 92.50, and 153.17 g, respectively (*p* < 0.05). Figure 8c–f show that the changes in elasticity, cohesiveness, chewiness, and resilience followed a pattern similar to that of hardness, with all exhibiting a significant downward trend (*p* < 0.05). After 48 h, the elasticity in the CK, R-1, R-2, and R-3 groups decreased from an initial value of 1.267 to 0.752, 0.508, 0.618, and 0.705, respectively (*p* < 0.05), and the cohesiveness decreased from 0.722 to 0.421, 0.331, 0.361, and 0.398 in the CK, R-1, R-2, and R-3 groups, respectively (*p* < 0.05). Similarly, the chewiness and resilience of the CK group remained the highest, while the R-1 group experienced the greatest decline. However, the trends in viscosity were different. As shown in Figure 8b, the viscosity of the test groups was higher than that of the CK group, and the R-1 group exhibited the most rapid changes. After 24 h, the viscosity values for the R-1, R-2, and R-3 groups were 14.458, 13.59, and 12.358 g·s, respectively, while that of the CK group remained below 12 g·s. The viscosity values in the CK, R-1, R-2, and R-3 groups significantly increased to 15.94, 23.57, 19.34, and 18.00 g·s at 48 h, respectively (*p* < 0.05). These alterations can be attributed to the denaturation and aggregation of muscle proteins at elevated temperatures, leading to progressive softening of the tissue structure [67,68].

### 3.8. Water Distribution Analysis

LF-NMR can quickly and non-destructively determine the water state and fluidity in aquatic products [69], and MRI can directly obtain visualization image information from the sample.

#### 3.8.1. LF-NMR Analysis

**Figure 9:**
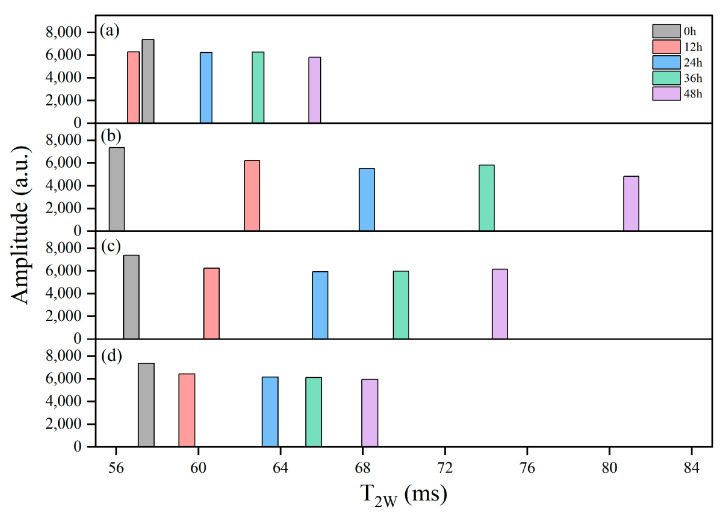
Distribution of single-component relaxation time (T_2w_) spectra for the CK (**a**), R-1 (**b**), R-2 (**c**), and R-3 (**d**) groups during delivery.

LF-NMR was employed to assess moisture status and mobility of the samples. As shown in Figure 9, T_2W_ generally increased with extended delivery time, and the groups experienced higher internal temperatures that corresponded to greater T_2W_ values. After 48 h, the T_2W_ of the R-1 and R-2 groups increased to 78.85 ms and 73.18 ms, respectively, compared to 64.98 ms in the CK group. In contrast, the R-3 group, which maintained a lower temperature, effectively slowed down water mobility, and exhibited a T_2W_ of 67.56 ms, closely resembling the CK group.

**Figure 10:**
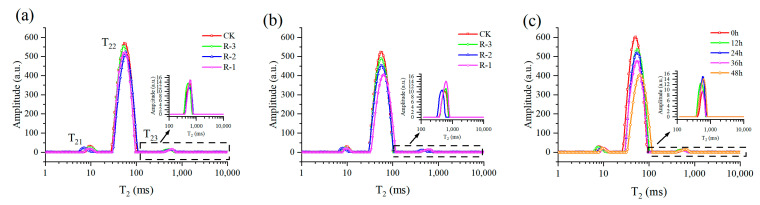
Distribution of multicomponent relaxation spectra (T_2_) for all four groups at 24 h (**a**) and 48 h (**b**), and for the R-1 group (**c**) during transport. CK is the control group, which was stored at 4 °C. R-1 is the group with one ice pack, R-2 is the group with two ice packs, and R-3 is the group with three ice packs; all were stored at 25 °C.

Figure 10 showed the multicomponent relaxation distribution (T_2_) of the four groups during delivery. Overall, three relaxation peaks were observed and named as peak T_21_, T_22_, T_23_, suggesting three water populations with different proton mobilities. The shorter the T_2_ is, the lower the mobility of water fraction is, and the faster relaxation is, or vice versa. The T_21_ peak (1–10 ms) was normally considered to represent the bound water, which was tightly associated with macromolecules. The protons of water trapped within the myofibrillar protein network were shown in the T_22_ peak, which is the dominant relaxing component, ranging from 10 to 100 ms. Meanwhile, the T_23_ peak (100–1000 ms) was related to the water outside the myofibrillar lattice and muscle cells [70].

Almost no shift occurred in the T_2_ relaxation distribution of the four groups at 24 h, as shown in Figure 10a; however, the transverse relaxation distribution exhibited a significant rightward shift, especially the T_22_ peak, after 48 h (Figure 10b). The rightward shift of the T_22_ peak was more pronounced in the R-1 and R-2 groups compared to the CK group, demonstrating the increased water mobility of samples experiencing higher temperatures. Taking the R-1 group, for instance (Figure 10c), with extension of delivery time, the T_2_ relaxation distribution gradually shifted to the right, and the amplitude of the T_22_ peak obviously decreased, suggesting an increase of water mobility and a reduction in the immobilized water population.

**Figure 11:**
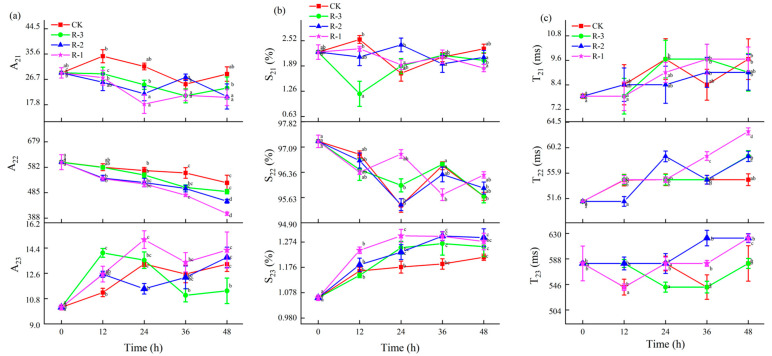
Changes in the relaxation parameters A_2i_ (**a**), S_2i_ (**b**), and T_2i_ (**c**) in the different groups during delivery. Different letters indicate significant differences between different times for the same treatment (*p* < 0.05). CK is the control group, which was stored at 4 °C. R-1 is the group with one ice pack, R-2 is the group with two ice packs, and R-3 is the group with three ice packs; all were stored at 25 °C.

The changes in the T_21_, T_22_, and T_23_ relaxation time constants, relative peak proportions (S_21_, S_22_, and S_23_), and peak areas (A_21_, A_22_, and A_23_) for the samples under different treatments were further analyzed, as shown in Figure 11. Changes in T_21_, A_21_, and S_21_ values were not obvious among the R-1, R-2, and R-3 groups compared to the CK group, suggesting that the binding strength between water and macromolecules remained relatively stable during delivery. However, as shown in Figure 11a,b, both the relative peak proportion (S_22_) and the peak area (A_22_) of the immobilized water showed overall decreasing trends along with the extension of delivery time, while S_23_ and A_23_, corresponding to free water, increased. Among the test groups, the R-3 group exhibited the smallest changes in S_2i_ and A_2i_, demonstrating a pattern more similar to the CK group, while the R-1 group demonstrated the most pronounced change. Taking A_22_ for instance, A_22_ significantly decreased from 601.35 to 522.62, 489.16, 452.30, and 404.85 in the CK, R-3, R-2, and R-1 groups after 48 h of transport, respectively (*p* < 0.05), while A_23_ significantly increased from 10.18 to 13.25, 11.35, 13.74, and 14.25 in the CK, R-3, R-2, and R-1 groups. The changes in A_22_ and A_23_ confirmed the dynamic changes in the water proton populations; that is, immobilized water transferred into free water during transport. As shown in Figure 11c, the T_22_ time constant for each treatment showed an increase. At 24 h, the T_22_ values of the R-1 and R-3 groups were similar to that of the CK group (54.79 ms), whereas the R-2 group had a slightly higher value of 58.73 ms. At the end of the transport period (48 h), the R-1 group demonstrated a pronounced change compared to the CK group, and the T22 values of the CK, R-1, R-2, and R-3 groups were 54.79 ms, 62.95 ms, 58.73 ms, and 58.79, respectively, showing an increase in water mobility. Compared to the CK and R-3 groups (541.59 ms), the T_23_ values for the R-1 and R-2 groups at 36 h were much higher, at 580.52 ms (R-1) and 622.26 ms (R-2), respectively. At 48 h, the T_23_ values for the R-1 and R-2 groups had all increased to 622.26 ms, which was significantly higher than that of the CK and R-3 groups (580.52 ms). This finding is consistent with a report by Sánchez-Valencia et al. [71], which noted that the relative abundance of immobilized water in hake decreased as delivery time increased. This phenomenon indicated that the trapped water was altered to free water during delivery, due to the destruction of myofibril structure [72], while sustained low temperatures hindered excessive water loss by slowing muscle fiber degradation.

#### 3.8.2. MRI Analysis

**Figure 12:**
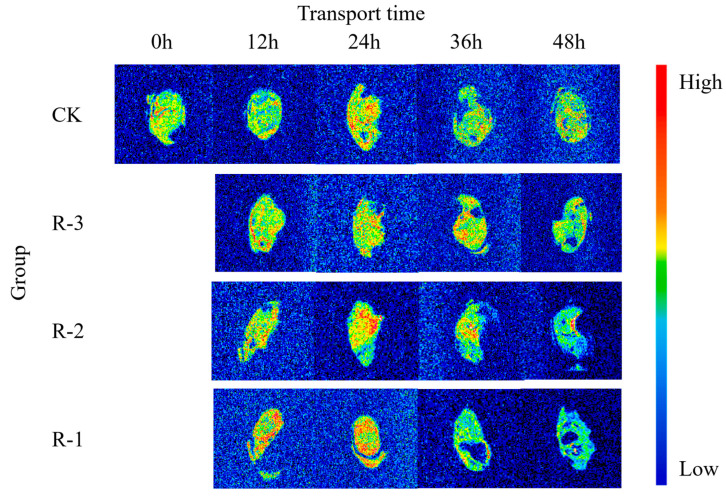
Proton density–weighted images of live clams in different packaging groups during delivery. CK is the control group, which was stored at 4 °C. R-1 is the group with one ice pack, R-2 is the group with two ice packs, and R-3 is the group with three ice packs; all were stored at 25 °C.

MRI was employed to visualize the water distribution, and the pseudo-color proton density-weighted images are displayed in Figure 12. The image contrast is dominated by the number of detectable protons in the voxel, and red areas indicate a higher density of hydrogen protons, signifying higher moisture content, while blue areas represent lower proton density, and therefore lower moisture content [73].

The T_2_-weighted image of CK appeared yellowish-green, reflecting a low proton signal and minimal water fluidity. However, visible color changes were observed as the transport time increased, and the variation was more pronounced for the groups with fewer ice packs. As shown in the pseudo-color images for groups R-1 and R-2 at 12 h, the water distribution inside the samples became uneven, and the enhanced proton signal resulted in more prominent red and yellow regions.

As the transport time extended from 12 to 24 h, the pseudo-color images showed a significant expansion in red regions across the sample sections. The R-1 and R-2 groups exhibited markedly larger red regions compared to the CK group, indicating a more pronounced rise in free water content and further highlighting the uneven water distribution throughout the sample. Particularly, the proton density in the 36–48 h pseudo-color images of the R-1 and R-2 groups showed a declining trend from the edge toward the center of the samples, and the colors progressively darkened to blue-green. During this period, significant water loss occurred in the muscle tissue, leading to a weakening of the signal intensity on the MRI images. This decrease may be due to the breakdown and denaturation of muscle proteins [74]. Meanwhile, as delivery time extended, formation of cavities in the pseudo-color images of the sample cross-sections was observed, especially in the samples exposed to higher temperatures. This might be related to the action of an endogenous protease that degrades isolated myofibrils, causing more rapid breakdown of the structures and thus the formation of the cavities [75]. Notably, the pseudo-color images of the R-3 group, which maintained a low temperature after 48 h, remained more similar to those of the CK group, suggesting that the quality of the R-3 group was better preserved.

#### 3.8.3. Correlation Analysis Between LF-NMR and Physicochemical Properties

**Figure 13:**
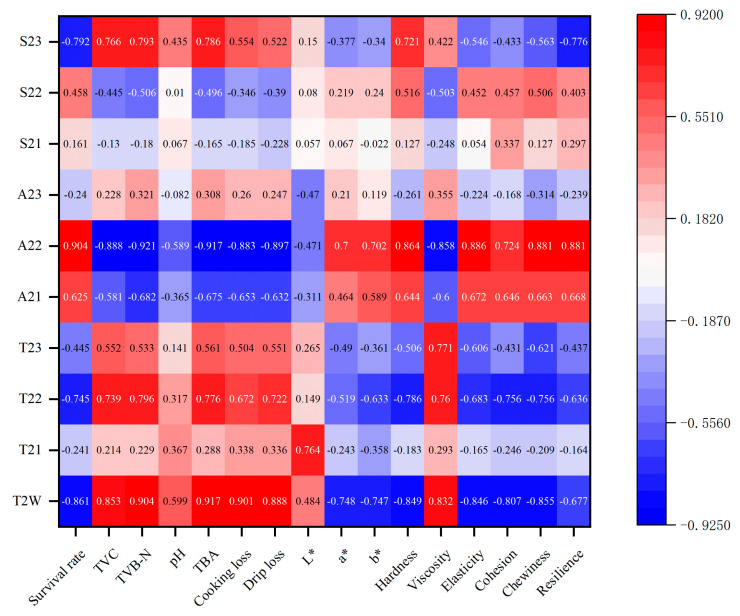
Pearson correlations between quality characteristics and LF-NMR relaxation.

Pearson correlation analysis was conducted to examine the relationship between LF-NMR measurements and physicochemical properties, and the results are presented in Figure 13 and Table A1. Positive or negative values indicate the direction of the correlation between the indicators, while the absolute values represent the strength of these correlations.

The survival rate was found to be negatively correlated with T_2W_ (r = −0.861 *), T_22_ (r = −0.745 *), and S_23_ (r = −0.792 *) (*p* < 0.05), but positively correlated with A_22_ (r = 0.904 **) (*p* < 0.01). Additionally, TVC, TVB-N, and TBA were significantly negatively correlated with A_22_ (r = −0.888 **, r = −0.921 **, and r = −0.917 **), while being positively or significantly positively correlated with T_2W_ (r = 0.853 *, r = 0.904 **, and r = 0.917 **) and S_23_ (r = 0.766 *, r = 0.793 *, and r = 0.786 *). Moreover, TBA exhibited a positive correlation with T_22_ (r = 0.776 *).

WHC showed a strong correlation with LF-NMR. Both cooking loss and drip loss were significantly positively correlated with T_2w_ (r = 0.901 **, r = 0.888 **) (*p* < 0.01) and negatively correlated with A_22_ (r = −0.883 **, r = −0.897 **) (*p* < 0.01). In addition, drip loss was positively correlated with T_22_ (r = 0.722 *) (*p* < 0.05).

With regard to texture properties, hardness exhibited significant positive correlations with A_22_ (r = 0.864 **) and S_23_ (r = 0.721 *) (*p* < 0.05 or *p* < 0.01). However, hardness was negatively correlated with T_2w_ (r = −0.849 *) and T_22_ (r = −0.786 *) (*p* < 0.05). Viscosity demonstrated a positive correlation with T_2W_ (r = 0.832 *), T_22_ (r = 0.760 *) and T_23_ (r = 0.771 *) (*p* < 0.05), while negatively correlating with A_22_ (r = −0.858 *) (*p* < 0.05). Elasticity showed a significant positive correlation with A_22_ (r = 0.886 **) (*p* < 0.01) and a negative correlation with T_2w_ (r = −0.846 *) (*p* < 0.05). Cohesion was negatively correlated with T_2w_ (r = −0.807 *) and T_22_ (r = −0.756 *) (*p* < 0.05). Chewiness was significantly positively correlated with A_22_ (r = 0.881 **) (*p* < 0.01) and negatively correlated with T_2w_ (r = −0.855 *) and T_22_ (r = −0.756 *) (*p* < 0.05). Resilience also demonstrated a significant positive correlation with A_22_ (r = 0.881 **) (*p* < 0.01) and a negative correlation with S_23_ (r = −0.776 *) (*p* < 0.05). In contrast, among the color indices, only the a* and b* values exhibited negative correlations with T_2W_ (r = −0.748 *, r = −0.747 *, respectively) (*p* < 0.05), while the L* value was positively correlated with T_21_. These findings are similar to those reported by Tan et al. [76] and Xie et al. [77], suggesting that LF-NMR can serve as a reliable tool for monitoring the physicochemical changes and quality deterioration of *R. philippinarum* during delivery.

## 4. Conclusions

The results from this study indicate that different packaging treatments significantly influence the quality of anhydrously preserved live *R. philippinarum* during disruption in the “last mile” of the cold chain. The study revealed significant variations in temperature field distribution according to the different packaging treatments, as assessed by COMSOL Multiphysics^®^. The R-3 group with three ice packs exhibited the lowest temperatures and restricted the movement of hot spots toward the center. Using different physicochemical indices to assess quality changes in live *R. philippinarum* with the three packaging treatments, we found that, with the extension of cold chain breakage time, the survival rate and sensory score of live *R. philippinarum* significantly decreased, while an increasing trend was observed in TVC, TVB-N, TBA, cooking loss, and drip loss. Fewer ice packs, which promotes a faster rise in temperature intensified changes in the above indices. In addition, the texture of live *R. philippinarum* deteriorated to different degrees; the hardness, elasticity, chewiness, cohesion, and resilience were significantly decreased (*p* < 0.05), whereas the viscosity was significantly increased (*p* < 0.05), although small changes occurred in elasticity and resilience. The a^*^ and b^*^ values significantly increased, whereas the L^*^ values decreased. After 48 h, accompanied by severe corruption, the R-1 group turned dark and yellowish. The change in color in the CK group was small, with a changing trend similar to that seen in the R-3 group. Moreover, the water state profile during delivery was characterized by LF-NMR and MRI. The relaxation characteristics of live *R. philippinarum* demonstrated that T_2w_ increased, and the T_22_ peak signal amplitude decreased and shifted to the right, indicating that the proportion of bound water decreased, while the proportion of free water increased. The overall change in the regularity of the CK and R-3 groups was small, contrary to that of the R-1 and R-2 groups, which was caused by maintaining a lower temperature. With a decrease in sample quality, the MRI pseudo-color images showed an expansion of red and yellow regions, followed by cavity formation due to tissue collapse and a decrease in proton density due to the high degree of water loss, causing the pseudo-color map to change to blue-green. The relaxation parameters assessed by LF-NMR exhibited good correlation with survival rate, microbial and classical physicochemical indices, color difference, and texture. These findings underscore the fact that using more ice packs, such as in the R-3 group, can not only maintain lower temperature but also reduce losses caused by “last mile” cold chain breakage, providing a reference for optimizing distribution conditions for live *R. philippinarum*.

## Figures and Tables

**Table 1:** Parameters of the phase change material (ice packs) used in the model.

Parameter Name	Value	Description
T_1→2_/K	273.15	Phase transition initiation temperature
∆T_1→2_/K	3.5	Phase transition temperature interval
L_1→2_/(kJ·kg^−1^)	333	Latent heat of phase transformation
T_ice_/K	268.15	Ice pack initial temperature
T_0_/K	293.15	Model initial temperature
T_content_/K	278.15	Content initial temperature

**Table 2:** Characteristics of the clams.

Characteristics	Value	Determination Method
Thermal conductivity, $\lambda_{f}$ (W·m^−1^·K^−1^)	0.4	Estimation from the mass fraction of water, protein, and fat [23]
$\mathrm{Density},\;\rho_{f}$ (kg·m^−3^)	1200	Calculation from the measured volume and weight
Thermal capacity, C_pf_ (J·kg^−1^·K^−1^)	3800	Experimentally measured

**Table 3:** RMSE coefficients of predicted and measured temperatures at different times.

Group	RMSE (°C)
R-1	0.97115
R-2	0.8858
R-3	0.81767

## Data Availability

The original contributions presented in the study are included in the article, further inquiries can be directed to the corresponding author.

## Appendix A

**Table A1:** Correlation equations between the LF-NMR relaxation characteristics and physicochemical and quality characteristics.

Index	Equation	R^2^
Survival rate	0.155 A_22_ − 45.054 S_23_ + 54.622	0.858
TVC	3.699 S23 − 0.027	0.900
TBARS	0.018 T_2W_ + 0.066 T_21_ + 0.514 S_23_ − 1.969	0.936
TVB-N	0.538 T_2W_ + 0.039 T_23_ + 7.315 S_23_ − 12.642	0.909
L*	0.194 A_21_ − 0.038 A_22_ + 0.067 T_23_ − 1.983 A_23_ + 47.248	0.848
b*	0.055 T_23_ + 0.302 A_23_ − 2.855 S_23_ + 1.989	0.857
Cooking loss	0.683 T_2W_ − 37.939	0.801
Hardness	−29.164 T_21_ − 440.284 S_23_ + 1108.064	0.951
Elasticity	−0.032 T_2W_ − 0.018 T_22_ + 0.002 T_23_ + 2.608	0.811
Cohesion	−0.011 T_2W_ + 0.155 S_21_ + 0.001 T_23_ + 0.048 A_23_ − 0.364 S_23_ + 0.097	0.810
Chewiness	0.247 A_22_ − 46.713 S_23_ + 66.359	0.804
Resilience	−0.003 T_2W_ − 0.031 S_21_ − 0.106 S_23_ + 0.506	0.834
